# Taking the Next Step in Obstetric Critical Care: Cardiac and Lung POCUS Training for Obstetric Residents

**DOI:** 10.24908/pocusj.v11i01.19976

**Published:** 2026-04-22

**Authors:** Celia A. Muoser, Avish Arora, Jay Im, Diana S. Wolfe

**Affiliations:** 1 Division of Maternal Fetal Medicine, Department of Obstetrics & Gynecology and Women's Health, Montefiore Medical Center/Albert Einstein College of Medicine, Bronx, NY, USA; 2 Department of Anesthesia, Montefiore Medical Center/Albert Einstein College of Medicine, Bronx, NY, USA

**Keywords:** Maternal Fetal Medicine, Obstetrics, Point-of-Care-Ultrasound, POCUS, Labour and Delivery, Residents

## Abstract

**Background:**

The importance of cardiopulmonary point of care ultrasound (POCUS) for the assessment of the acute obstetric patient is increasingly recognized. Obstetricians are well-versed in ultrasound and may be able to perform POCUS with brief training.

**Objective:**

To implement and assess a hands-on basic training program in cardiopulmonary POCUS for obstetric trainees.

**Methods:**

Obstetric residents participated in a basic training program for cardiopulmonary POCUS run by maternal-fetal medicine and cardiac anesthesia specialists. A pre- and post-survey on confidence and self-perceived likelihood to utilize learned skills was administered.

**Results:**

Twenty-seven trainees participated in our program. Participants showed improved confidence in obtaining cardiac and lung views, as well as using the cardiac probe. They felt more comfortable making clinical decisions based on POCUS findings and reported they were more likely to use POCUS on obstetric patients.

**Conclusions:**

Our study demonstrated the feasibility of a POCUS training program for obstetric residents.

## Introduction

Cardiopulmonary point of care ultrasound (POCUS) has emerged as a useful and efficient imaging modality for timely diagnosis and enhanced medical decision-making. In 2004, the American Institute of Ultrasound in Medicine (AIUM) introduced the idea of using the “ultrasound as a stethoscope” or adjunct to a clinician's bedside clinical exam [1]. Subsequently, guidelines for POCUS exams have been established by the Society of Critical Care Medicine [2,3]. As opposed to conventional formal ultrasound, POCUS is conducted in a focused fashion with emphasis on views and findings that can guide differential diagnosis and treatment in medically acute patients.

POCUS has been used extensively in emergency medicine and critical care settings [4,5]. More recently, it has been utilized by internal medicine, family medicine, and pediatric providers [6–8]. The importance of cardiopulmonary POCUS for assessment of the acute obstetric patient is increasingly recognized, particularly in the setting of acute cardio-respiratory failure or to assist with peripartum fluid management [9–11]. While critical care or anesthesia providers may be available for consultation, obstetricians are also well versed in ultrasound as an imaging modality and may be able to perform cardiopulmonary POCUS with brief training [12,13]. Additionally, an obstetric ultrasound probe is readily available on labor and delivery units, making POCUS for the obstetric provider a quick and feasible tool while awaiting additional imaging or subspecialist assistance. The American College of Obstetricians and Gynecologists (ACOG) recently acknowledged the role of POCUS in pregnancy [14]. Leaders in the field of maternal-fetal medicine issued a call for maternal POCUS techniques and skills to be incorporated into obstetric training curricula [15]. In obstetric care, cardiopulmonary POCUS has particular clinical value for rapid evaluation of dyspnea, assessment of peripartum cardiomyopathy and cardiopulmonary complications of severe preeclampsia, identification of pulmonary edema, and guidance of volume status during hemorrhage resuscitation [9–11,15].

Previous basic training programs for POCUS in internal medicine and family medicine residencies have shown that proficiency and competence in cardiopulmonary POCUS can be achieved, particularly when hands-on training is used [16–18]. With the proposed benefit of expedited diagnosis and treatment in the acute labor and delivery setting, obstetrics should also consider the feasibility of such training. This study aimed to implement and assess a hands-on basic training program in cardiopulmonary POCUS for obstetric residents.

## Methods

**Figure 1.**
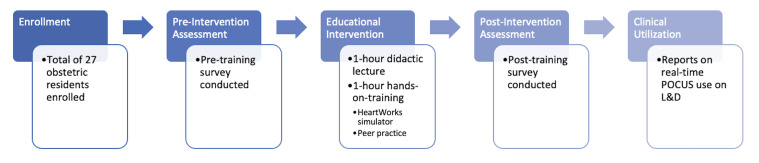
Study overview. L&D, learning and development; POCUS, point of care ultrasound.

Obstetric residents at a large, academic, urban medical center (Montefiore Medical Center/Albert Einstein College of Medicine in the Bronx, NY) participated in a basic training program for cardiopulmonary POCUS. This was a pre-post intervention study with survey assessment of participants before and after the training (Figure 1). Sessions were conducted in small groups from April 2023 through March 2024. Participation was voluntary, and informed consent was obtained from all participants. Inclusion criteria for participation included current enrollment in our Obstetrics & Gynecology residency program (post-graduate years 1–4) and consent to participate, while exclusion criteria included any prior formal cardiopulmonary POCUS training.

**Figure 2.**
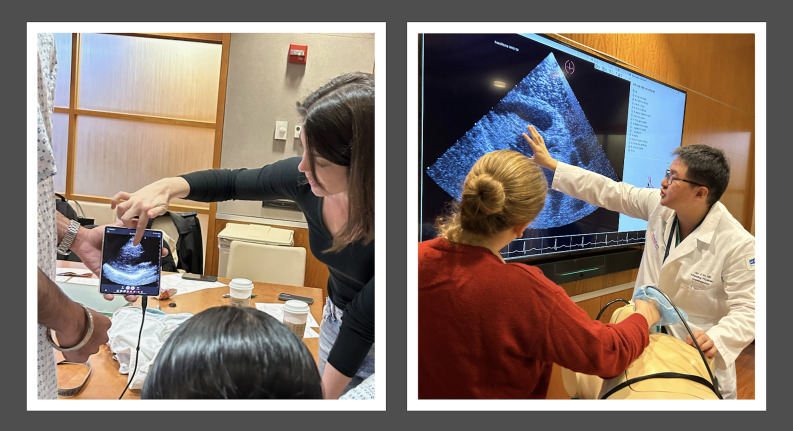
Participants in mannequin simulation and hands-on point of care ultrasound (POCUS) training.

The program consisted of a one-hour lecture with educational handouts (Supplementary Material S1), followed by a simulation session with an ultrasound-compatible mannequin and hands-on practice of lung and heart views (Figure 2). The training program was run by maternal-fetal medicine and cardiac anesthesia specialists with prior clinical POCUS experience. All participants had the opportunity to use the HeartWorks (Surgical Science Sweden AB, Göteborg, Sweden) mannequin for cardiac views, as well as practice live cardiac and pulmonary views on one another. Participants of all post-graduate years received the same training.

Basic knowledge of ultrasound is already part of the obstetric skill set, so training focused on the use of the cardiac probe and obtaining specific heart and lung views and interpretation. Five views commonly used in cardiac and pulmonary ultrasonography were taught as the foundation of cardiac POCUS. These views are adapted from those previously outlined in the CLUE (Cardiac Limited Ultrasound Examination) and RACE (Rapid Assessment of Competency in Echocardiography) protocols used for established POCUS training protocols [7,19]:

1. Parasternal – long and short axis
2. Apical – 4 chamber view
3. Subcostal – 4 chamber view and inferior vena cava
4. Lung apex – left and right
5. Lung base – left and right

The objective of the training was for obstetric residents to be able to obtain views adequate for image interpretation and to identify pathologies that may change acute medical management in the labor and delivery unit, including abnormal cardiac dilation and ventricular function, pulmonary edema, pericardial effusion, and intravascular volume depletion. The goal of the program was to build foundational POCUS awareness and build pattern recognition. Only gross abnormalities were expected to be recognized with POCUS as a tool to escalate care, while complex or structural cardiac disease was not expected to be diagnosed.

A survey on level of confidence and self-perceived likelihood to utilize learned skills was administered before and after the training session. The survey collected responses to statements on a 5-point Likert scale ranging from 1 (not at all confident/likely) to 5 (very confident/likely). The Wilcoxon Signed Rank Sum test was used to compare pre- and post-training responses and compensate for non-normality. A p-value of <0.05 was considered statistically significant. A subset of participants answered additional questions about their experience with the use of POCUS in real-time on the labor and delivery unit.

## Results

Twenty-seven obstetric residents participated in our POCUS training program. All residents completed both the lecture and simulation/hands-on portions of the training. Residents of all experience levels were included and completed the training. Seven were post-graduate year 4 (PGY4), eleven were PGY3, four were PGY2, and five were PGY1.

For the pre-training confidence statements, participants were most confident in the use of the obstetric probe and the appropriate clinical setting in which to use POCUS based on median scaled responses. They were not at all confident in using the cardiac probe or obtaining either cardiac or pulmonary views (Table 1).

**Table 1.** Self-confidence and perception survey. POCUS, point of care ultrasound. 5-point Likert scale ranging from 1 (not at all confident/likely) to 5 (very confident/likely).

Statement	Pre-training Median	Post-training Median	Signed Rank S Statistic	p-value	Median Change	Range of Change
I am confident about the appropriate clinical setting in which to use POCUS	3	4	162.5	<0.0001	1	3
I am confident in using an obstetric probe	4	4	0	1	0	2
I am confident in using a cardiac probe	1	3	162.5	<0.0001	2	3
I am confident obtaining cardiac views	1	3	175.5	<0.0001	2	4
I am confident obtaining pulmonary views	1	4	175.5	<0.0001	2	4
I am confident in making clinical decisions based on POCUS findings	2	3	133.5	<0.0001	2	5
I am likely to use POCUS on the labor and delivery unit for a pregnant or peripartum patient with cardiopulmonary symptoms	2	4	142	<0.0001	2	5
I am likely to teach another obstetric provider POCUS	1	3	114.5	<0.0001	1	5

Significant improvements in confidence were seen in all survey responses post-training, except confidence with the obstetric probe. Participants gained confidence in obtaining both cardiac and pulmonary views. Additionally, participants were significantly more likely to feel they could use POCUS on the labor and delivery unit after the training, and significantly more likely to feel they could teach another provider POCUS (Table 1).

A subset of 10 participants was asked about subsequent use of POCUS on the labor and delivery unit. Four out of the ten reported using POCUS in real-time for patient care after the training program. Three out of those four reported that POCUS decreased the time to rule out or diagnose cardiopulmonary disorders in a clinical setting. Additional work-up prompted by POCUS use included chest X-rays and computed tomography imaging. Interventions prompted by POCUS use included critical care evaluation, diuretic administration, and discontinuation of intravenous magnesium.

## Discussion

This study demonstrated the feasibility of a basic training program for cardiopulmonary POCUS for obstetric residents. Through a structured curriculum, we were able to increase confidence in using bedside ultrasound to obtain cardiac and lung views, similar to prior studies done in other medical specialties [18]. This is the inception of an established universal program to train obstetric trainees, which is an ultimate goal for the obstetric community. We found that residents were able to adapt technical ultrasound skills already in their repertoire with the cardiac probe. Our residents have had some exposure to POCUS on the labor and delivery unit as performed by maternal-fetal medicine providers, so it is not surprising that there was already some comfort level about the appropriate clinical setting in which to use POCUS—for example, a preeclamptic patient with shortness of breath. Our training sessions allowed them to take the next step and develop their own acquisition and interpretation skills for cardiopulmonary POCUS, increasing the likelihood that they would feel comfortable using these skills for critically ill obstetric patients. Additionally, we found that some residents were able to employ the skills they learned in training in patient care.

To our knowledge, this is the first study to describe and assess a POCUS training program for obstetric residents. With the recent call for maternal POCUS technique and skills to be incorporated into obstetric training curriculum [15], we expect further programs and research in this emerging topic in the coming years. Our program's strengths include standardization of training for small groups of residents within a single program, incorporation of simulation and hands-on training, and interdisciplinary collaboration between maternal-fetal medicine and cardiac anesthesiology.

Limitations of the study include the small number of participants, a lack of a validated survey instrument, and a lack of objective measurement of POCUS skills. Our survey instrument was adapted from prior similar studies aimed at teaching POCUS to residents [17]. However, we were unable to identify a standardized or validated survey tool to use in this setting. Additionally, in this first phase of our program, we could not test the proficiency and accuracy of obstetric residents in obtaining images. To fully evaluate a training program, an objective assessment of image acquisition, image quality, and diagnostic accuracy is vital. Studies in internal and family medicine have assessed resident-obtained images after POCUS training to help determine competency [16,18]. This is something we hope to implement and examine in the future.

Furthermore, a formalized assessment of clinical decisions based on POCUS use is needed. As described by Easter et al. in a 2023 expert review, POCUS can be used to guide clinical management in the setting of acute obstetric emergencies, assist with decisions for fluid management, and help to narrow a differential diagnosis [15]. Future studies should examine the use of POCUS by obstetric trainees under faculty supervision in labor and delivery units over time and ideally assess the time to clinical intervention and final diagnosis both with and without POCUS use.

Our program, as described here, is just a starting point for introducing cardiopulmonary POCUS into the obstetric training curriculum. We hope to continue training residents at our institution and begin to examine objective measures of proficiency after completing the training. Additionally, we need to examine the usefulness of the resident training on care provided to critically ill patients on the labor and delivery unit in a meaningful way. Perhaps examining the time to diagnosis of cardiopulmonary disorders or intervention with and without POCUS can help to demonstrate the utility of such training in the obstetric setting. Ideally, our program curriculum and materials could be adapted for use with a larger audience of obstetric providers and trainees.

## Conclusion

Our study demonstrated the feasibility and benefits of a cardiopulmonary POCUS training program for obstetric residents. This is a skill that is valuable for expedited diagnosis and treatment of acute obstetric patients. Further work should objectively assess the proficiency of obstetric trainees with POCUS and evaluate its use in the labor and delivery setting.
