# Supplementary figures and images for: Taking the Next Step in Obstetric Critical Care: Cardiac and Lung POCUS Training for Obstetric Residents
Source: POCUS J. 2026 Apr 22;11(1):22–6. doi: 10.24908/pocusj.v11i01.19976 (PMC13161777; doi:10.24908/pocusj.v11i01.19976)

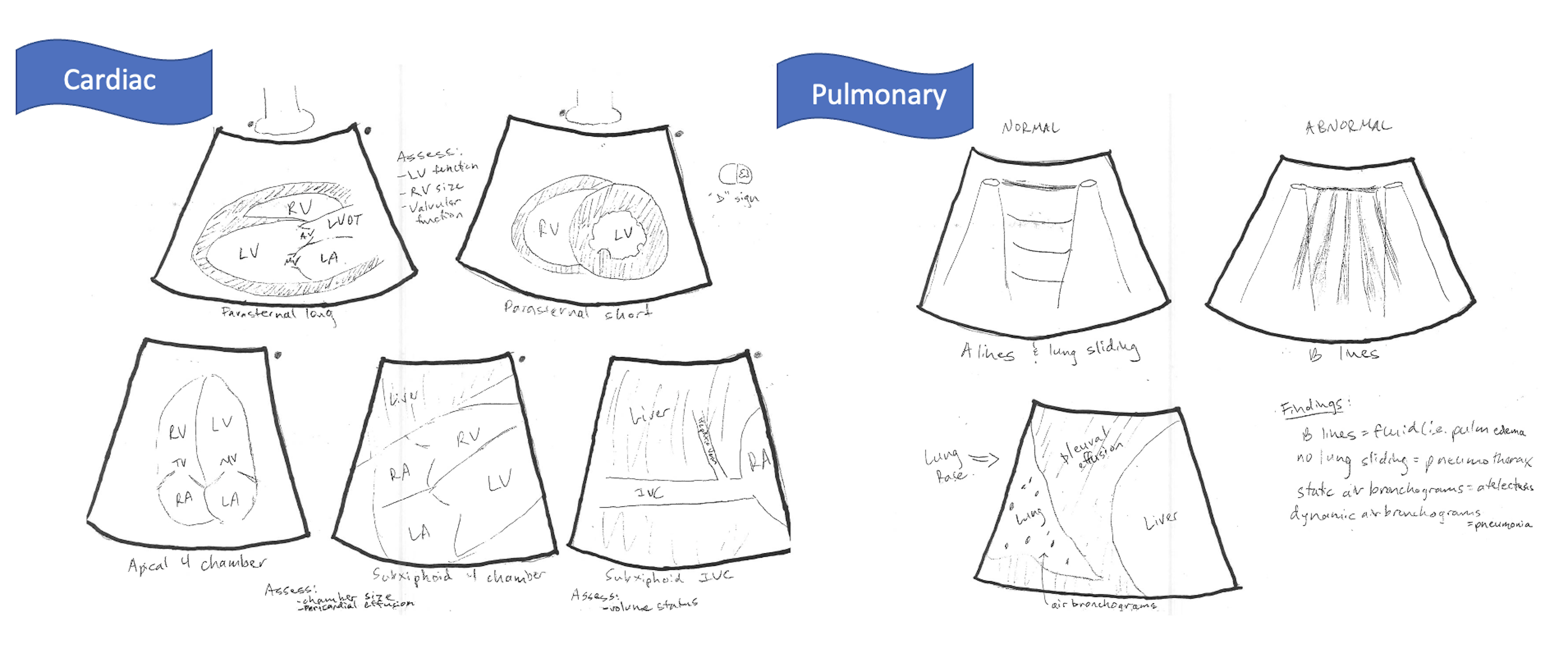

Supplement: Supplementary file 1 [file pocusj-11-01-19976-s001.tiff]
